# Identifying a commercially-available 3D printing process that minimizes model distortion after annealing and autoclaving and the effect of steam sterilization on mechanical strength

**DOI:** 10.1186/s41205-020-00062-9

**Published:** 2020-04-15

**Authors:** Joshua V. Chen, Kara S. Tanaka, Alan B. C. Dang, Alexis Dang

**Affiliations:** 1 Department of Orthopaedic Surgery, University of California, San Francisco, CA USA; 2 Department of Surgery, Orthopaedic Section, San Francisco VA Health Care System, San Francisco, CA USA

**Keywords:** 3D printing, 3D printing materials, Additive manufacturing, Annealing, Autoclave, Medical devices, Optimization, Sterilization, Surgical instruments, Polylactic acid

## Abstract

**Background:**

Fused deposition modeling 3D printing is used in medicine for diverse purposes such as creating patient-specific anatomical models and surgical instruments. For use in the sterile surgical field, it is necessary to understand the mechanical behavior of these prints across 3D printing materials and after autoclaving. It has been previously understood that steam sterilization weakens polylactic acid, however, annealing heat treatment of polylactic acid increases its crystallinity and mechanical strength. We aim to identify an optimal and commercially available 3D printing process that minimizes distortion after annealing and autoclaving and to quantify mechanical strength after these interventions.

**Methods:**

Thirty millimeters cubes with four different infill geometries were 3D printed and subjected to hot water-bath annealing then immediate autoclaving. Seven commercially available 3D printing materials were tested to understand their mechanical behavior after intervention. The dimensions in the X, Y, and Z axes were measured before and after annealing, and again after subsequent autoclaving. Standard and strength-optimized Army-Navy retractor designs were printed using the 3D printing material and infill geometry that deformed the least. These retractors were subjected to annealing and autoclaving interventions and tested for differences in mechanical strength.

**Results:**

For both the annealing and subsequent autoclaving intervention, the material and infill geometry that deformed the least, respectively, was Essentium PLA Gray and “grid”. Standard retractors without intervention failed at 95 N +/− 2.4 N. Annealed retractors failed at 127.3 N +/− 10 N. Autoclave only retractors failed at 15.7 N +/− 1.4 N. Annealed then autoclaved retractors failed at 19.8 N +/− 3.1 N. Strength-optimized retractors, after the annealing then autoclaving intervention, failed at 164.8 N +/− 12.5 N.

**Conclusion:**

For 30 mm cubes, the 3D printing material and infill geometry that deformed the least, respectively, was Essentium PLA and “grid”. Hot water-bath annealing results in increased 3D printed model strength, however autoclaving 3D prints markedly diminishes strength. Strength-optimized 3D printed PLA Army-Navy retractors overcome the strength limitation due to autoclaving.

## Background

3D printing is currently used in the medical field for a wide variety of purposes, including printing patient-personalized anatomical models to guide surgeons preoperatively, creating in-house anatomical models for medical student and resident training, and printing surgical instruments, prostheses, and implants [1–7]. 3D printed models have already been adopted to plan surgeries in fields including, but not limited to, cardiothoracic, craniomaxillofacial, hepatic, neonatal, neurological, ophthalmologic, orthopaedic, and plastic surgery [8–19]. Therefore, the accuracy of 3D printed models becomes exceedingly important. Equally as important, however, is understanding the behavior of these models after steam sterilization for use in the sterile surgical field. It has been previously understood that subjecting polylactic acid (PLA) to steam sterilization severely weakens PLA [20]. Although there exists literature suggesting that 3D printed models minimally deform after autoclave sterilization, there is much that is unknown about improving the mechanical strength of these models [21].

Current literature supports that annealing heat treatment of PLA 3D printed models increases the models’ crystallinity, thereby increasing their mechanical strength [22–25]. Therefore, the next step would be to identify an optimal 3D printing material which, when subjected to both heat treatment and steam sterilization, deforms the least and remains strong enough to be safely used in the operating room.

The goal of this paper is to identify a suitable commercially-available fused deposition modeling (FDM) 3D printing material that deforms minimally after hot water-bath annealing and autoclaving to create surgical instruments and anatomical models for use in the sterile field and to evaluate the effects of these interventions on mechanical strength. We assessed different physical properties of the 3D printing filament: ability to withstand annealing and autoclaving with minimal distortion, mechanical strength, and how infill pattern affects model stability throughout the heating process.

## Methods

**Fig. 1:**
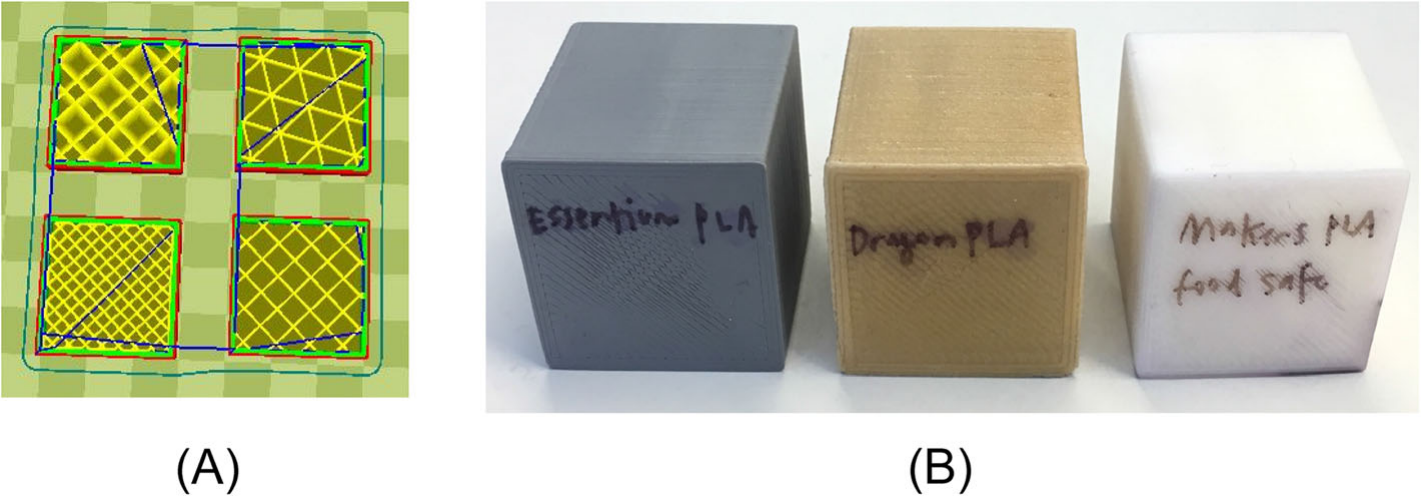
**a** Infill geometries clockwise beginning from top-left: tetrahedral, triangles, grid, zig-zag and **b** 3D printed cubes

Four 30 mm cubes with different infill geometries were designed with the software Tinkercad™ (Autodesk®, Inc.), and were exported as a standard tessellation language (STL) file. The STL file was then imported into Ultimaker Cura LulzBot® Edition software (Ultimaker B.V., version 21.08, Aleph Objects, Inc.), where adjustments for wall thickness of 1.5 mm and different infill geometries, “tetrahedral”, “triangles”, “zig-zag”, and “grid”, were selected (Fig. 1). The cubes were printed 20% infill. Once print settings were satisfactory, the design was exported as a g-code, a set of spatial instructions that guide the 3D printer, and uploaded onto the LulzBot® Mini 3D printer (Aleph Objects, Inc.).

The four cube designs were printed simultaneously with 0.38 mm layer height utilizing a 0.5 mm printhead nozzle. Print bed and printhead nozzle temperatures were selected according to manufacturer specifications for each print material using the highest recommended nozzle temperatures, defaulting to 60 °C for bed temperature (Table 1). Higher temperatures have been shown to optimize layer adhesion and strength for FDM printing. Filament materials were selected based on consumer accessibility.

**Table 1:** Manufacturer temperature (°C) recommendations for FDM 3D printing materials

3D Printing Material	Nozzle T	Heated Bed T
colorFabb Woodfill	210	60
Dragons Metallic PLA All That Glitters Gold	235	60
Essentium PLA Gray	240	60
Maker Series PLA, Food Safe, FDA OK, Clear	240	60
Maker Series PLA White HOT White	235	60
Proto-Pasta HTPLA White	240	60
Raptor Series PLA HD Vivid Blue	235	60

The seven materials tested were colorFabb Woodfill (ColorFabb BV, Netherlands), Dragons Metallic PLA in All That Glitters Gold (Maker Geeks, USA), Essentium PLA in Gray (Essentium Materials LLC, USA), Maker Series PLA in Food Safe FDA OK Clear (MatterHackers, Inc., USA), Maker Series PLA in White HOT White, Proto-Pasta HTPLA in White (Protoplant, Inc., USA), and Raptor Series PLA in HD Vivid Blue (Maker Geeks, USA).

The dimensions of each cube were measured using digital calipers at midline along the X, Y, and Z axes, where Z is the axis perpendicular to the build plate. All measurements were collected by a single operator to reduce bias and variability; calipers were zeroed between each set of cubes. Baseline measurements were collected after cubes were printed and again after each intervention.

Cubes were subjected to a hot water-bath annealing treatment using an 800 W Strata Home sous vide circulating precision cooker (Monoprice, Inc., Brea, CA) for 30 mins at 100 °C. The cubes were removed from the hot water-bath and allowed to cool to room temperature without interference. The X, Y, and Z dimensions of the cubes were measured again to quantify deformation and calculate percent changes, a positive percent change indicating expansion and a negative percent change indicating shrinkage. In order to quantify distortion in either direction, we took the absolute value of these percentages. Subjective observations were noted such as spherical “balloon-like” expansion. We also analyzed whether certain materials consistently expanded or contracted in every axes.

Following the hot water-bath annealing treatment, the cubes were placed in Chex-all® II Instant Sealing autoclave sterilization pouches (Propper Manufacturing Co. Inc., USA) and subjected to surgical grade autoclaving in a Tuttnauer 2540 M autoclave (Tuttnauer USA) for 45 mins at 134 °C and a pressure of 375 PSI. Autoclave indicator tape (Propper Manufacturing Co. Inc., USA) was used to verify that appropriate sterilization conditions were met. The packages were removed from the autoclave and the cubes were allowed to cool to room temperature. Each cube was measured for additional distortion and subjective observations were noted. While the CDC minimum recommended time for steam sterilization is 30 mins at 121 °C for the effective sterilization of wrapped healthcare supplies, in our study, we selected more challenging parameters of 45 mins at 134 °C for single-use custom devices and 3D printed anatomical models [26].

While gamma irradiation and ethylene oxide sterilization are useful for heat sensitive or moisture sensitive instruments or devices, steam sterilization was selected for this study, as it is low-cost and readily available in environments where 3D printed tools are expected or potentially may be used.

To determine which 3D printing material deforms the least, the hot water-bath annealing treatment measurements were summed to quantify the absolute value of the percent change in the X, Y, and Z direction for each cube. We averaged these quantities for all four infill patterns for each material to quantify an average absolute percent deformation to objectively determine which 3D printing material deforms the least in a hot water-bath. This was repeated for the autoclave measurements, where the absolute values of percent change in all three axes were summed for each cube, and then averaged across all infill geometries for each material.

To determine which infill geometries deformed the least, we again summed the absolute value of the percent change in the X, Y, and Z direction for each cube, and then averaged the values for each of the seven materials, each printed using the same infill pattern. The same was done for the autoclave data.

The X, Y, and Z dimension percent changes for each material were averaged for both the hot water-bath heat treatment and autoclave intervention in order to analyze if and how distortion varies between the respective axes.

**Fig. 2:**
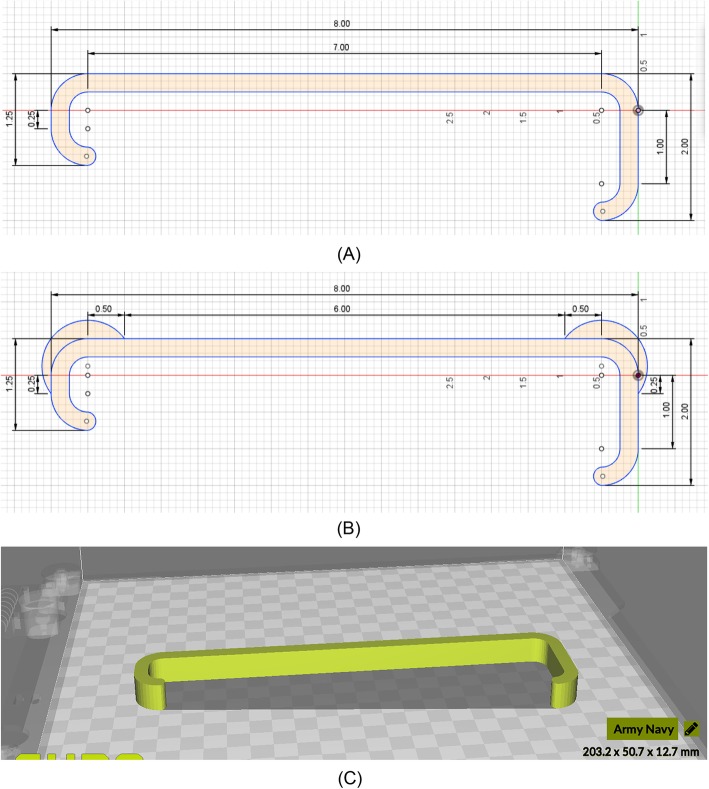
**a** Standard Army-Navy retractor and **b** strength-optimized Army-Navy retractor designs in inches created in AutoDesk® Fusion 360™ obtained from Chen et al. **c** Retractor orientation on the build plate to eliminate need for support material

We determined the best candidate 3D printing material and infill geometry from this analysis, Essentium PLA Gray filament and “grid” infill, respectively, and printed standard surgical Army-Navy retractors to test the mechanical strength of PLA following annealing and autoclaving interventions, both independently and together. Standard and strength-optimized Army-Navy retractor designs created by Chen et al. in Autodesk® Fusion 360™ were used, obtained as STL files (Fig. 2a, b) [27]. The annealing intervention was submersion of the retractors in a 100 °C water-bath for 30 mins, and the autoclaving intervention was autoclaving the retractors for 45 mins at 134 °C and a pressure of 375 PSI.

**Fig. 3:**
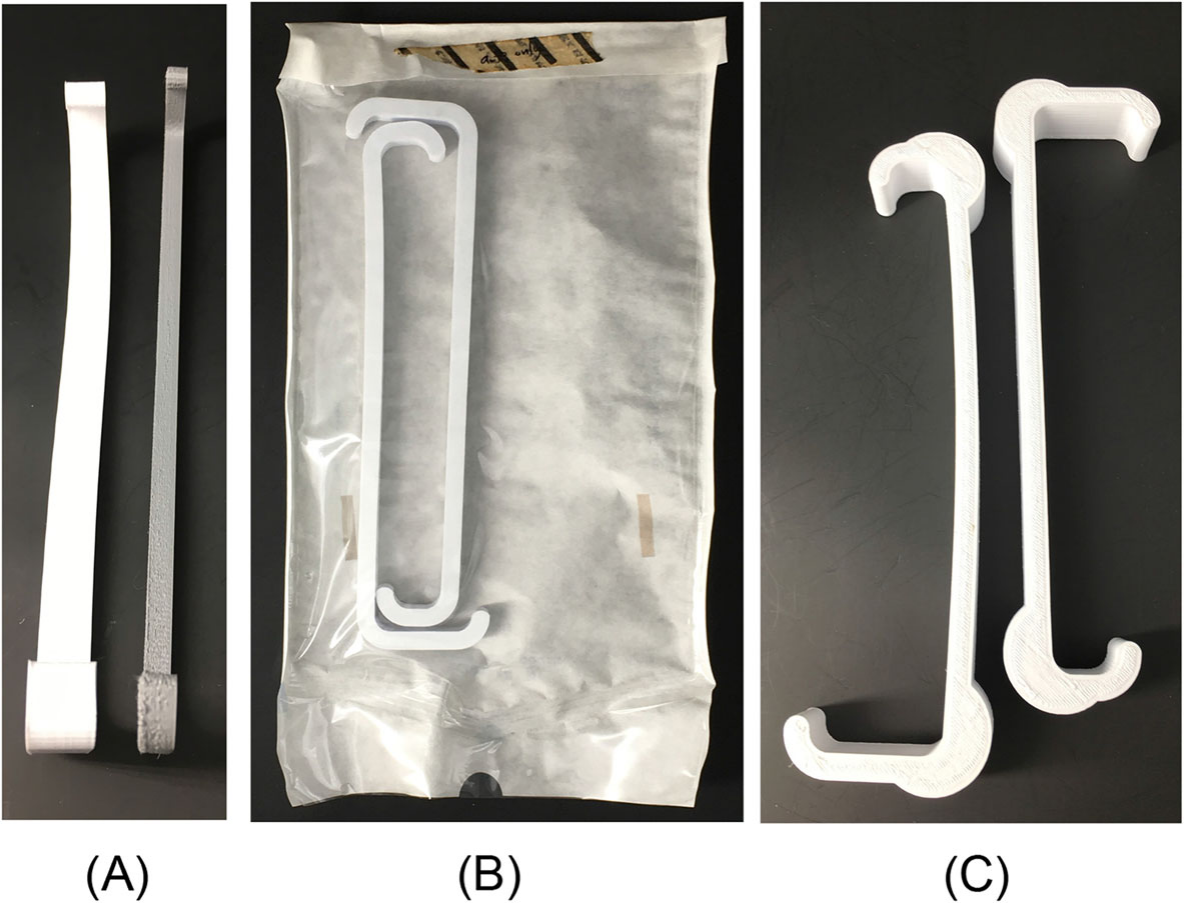
**a** Standard retractors warping after hot water-bath annealing and **b** after autoclaving. **c** Strength-optimized retractor without intervention (right) and warping after hot water-bath annealing (left)

Each STL file was imported into the Ultimaker Cura LulzBot Edition software and oriented on the print bed such that no support material is required (Fig. 2c). In order to improve the accuracy of mechanical strength measurements, we choose the infill pattern that minimizes distortion to reduce random error introduced through warping (Fig. 3). The infill pattern “grid”, which was found to minimize distortion after interventions, at 20% infill was selected. The design was then exported as a g-code and uploaded onto the LulzBot® Mini 3D printer. The extruder temperature was set to 235 °C.

Specified by Chen et al., the print settings for strength-optimized 3D printed PLA Army-Navy retractors were 30% infill, 3 perimeters, 0.25 in. thickness, 0.75 in. width, “triangles” infill geometry and reinforced joints, which optimizes retractor strength but does not aim to minimize deformation [27]. Strength-optimized retractors were subjected only to the annealing then autoclaving intervention to determine whether these 3D printed surgical retractors can remain robust even after steam sterilization, as we would find that annealing prior to autoclaving confers a strength advantage.

Twenty-four Essentium PLA Gray filament standard retractors using 20% “grid” infill geometry were printed and randomly placed into one of four groups: control, annealing only intervention, autoclaving only intervention, or annealing then autoclaving intervention. Six Essentium filament strength-optimized retractors were printed, annealed, and autoclaved to test whether they maintain structural integrity for use in the operating room.

These retractors were pulled until complete physical breakage using an FGS-1000H manual turn-wheel force test stand and FG-3009 digital force gauge (Nidec-Shimpo Corporation) such that the long arm of the retractor was placed on the strap and the short arm of the retractor was placed on the force gauge hook for consistency. The maximum force withstood was then collected to determine whether the interventions created differences in mechanical strength.

## Results

After hot water-bath annealing for 30 mm cubes, the material that deformed the least was Essentium PLA Gray, and the material that deformed the most was Maker Series PLA White HOT White (Table 2).

**Table 2:** Quantifying absolute deformation in 30 mm cubes across 3D printing materials after annealin

3D Printing Material	Average Absolute Percent Deformation Across All Infill Geometries
Essentium PLA Gray	1.601%
Proto-Pasta HTPLA White	2.058%
colorFabb Woodfill	3.414%
Raptor Series PLA HD Vivid Blue	5.584%
Dragons Metallic PLA All That Glitters Gold	6.587%
Maker Series PLA, Food Safe, FDA OK, Clear	10.702%
Maker Series PLA White HOT White	17.545%

After hot water-bath annealing for 30 mm cubes, the infill that deformed the least was “grid”, and the infill pattern that deformed the most was “zig-zag” (Table 3).

**Table 3:** Quantifying absolute deformation in 30 mm cubes across infill geometries after annealing

Infill Geometries	Average Absolute Percent Deformation Across All 3D Printing Materials
Grid	5.193%
Triangles	6.407%
Tetrahedral	7.710%
Zig-zag	7.827%

After both annealing then autoclaving for 30 mm cubes, the material that deformed the least was Essentium PLA Gray. The material that deformed the most was Maker Series PLA White HOT White (Table 4).

**Table 4:** Quantifying absolute deformation in 30 mm cubes across 3D printing materials after annealing then autoclaving

3D Printing Material	Average Absolute Percent Deformation Across All Infill Geometries
Essentium PLA Gray	1.637%
Proto-Pasta HTPLA White	1.799%
Raptor Series PLA HD Vivid Blue	6.183%
Dragons Metallic PLA All That Glitters Gold	6.345%
colorFabb Woodfill	6.363%
Maker Series PLA, Food Safe, FDA OK, Clear	10.468%
Maker Series PLA White HOT White	17.711%

After both annealing then autoclaving for 30 mm cubes, the infill pattern that deformed the least was “grid”, and the infill pattern that deformed the most was “tetrahedral” (Table 5).

**Table 5:** Quantifying absolute deformation in 30 mm cubes across infill geometries after annealing then autoclaving

Infill Geometries	Average Absolute Percent Deformation Across All 3D Printing Materials
Grid	5.459%
Triangle	6.633%
Zig-zag	8.377%
Tetrahedral	8.391%

In averaging the absolute values of the percent distortion in the X, Y, and Z direction across all 3D printing materials after hot water-bath annealing and again after autoclaving, it was found that distortion in the X and Y direction was comparable and that distortion primarily occurs in the Z axis, the axis perpendicular to the build plate (Table 6). The distortion in the Z direction is approximately twice the magnitude of distortion in either the X or Y direction.

**Table 6:** For 30 mm cubes, consistent across both interventions, distortion in the Z axis is approximately twice that of distortion in either X or Y axes

	Water Bath Annealing	Annealing then Autoclave
**Average absolute percent distortion in X axis**	1.677%	1.712%
**Average absolute percent distortion in Y axis**	1.628%	1.752%
**Average absolute percent distortion in Z axis**	3.480%	3.751%

All subjective observations were tabulated, most of which arose in cubes printed from a variant of the Maker Series PLA filament and having "tetrahedral" infill geometry (Table 7).

**Table 7:** All subjective observations noted in this study

3D Printing Material	Infill Geometry	Subjective Observation (Water Bath Annealing)	Subjective Observation (Autoclave)
Proto-Pasta HTPLA White	Tetrahedral	No observed subjective deformity	Mild Wave
Maker Series PLA, Food Safe, FDA OK, Clear	Triangles	Mild Balloon	No observed subjective deformity
Dragons Metallic PLA All That Glitters Gold	Tetrahedral	Balloon	Balloon
Maker Series PLA, Food Safe, FDA OK, Clear	Tetrahedral	Balloon	Balloon
Maker Series PLA White HOT White	Grid	Balloon	Mild Balloon
Maker Series PLA White HOT White	Tetrahedral	Balloon	Balloon
Maker Series PLA, Food Safe, FDA OK, Clear	Zig-zag	Balloon	Balloon
Maker Series PLA White HOT White	Triangles	Balloon	Balloon
Maker Series PLA White HOT White	Zig-zag	Balloon	Balloon

The material “Maker Series PLA White HOT White” is the only material to consistently expand in every axis, regardless of the infill geometry or intervention. All other materials in this study had varying degrees of both expansion and contraction depending on the infill geometry. Expansion or contraction in a certain axis after annealing generally, but not always, predicted the same direction of distortion after subsequent autoclaving (Additional file 1).

**Fig. 4:**
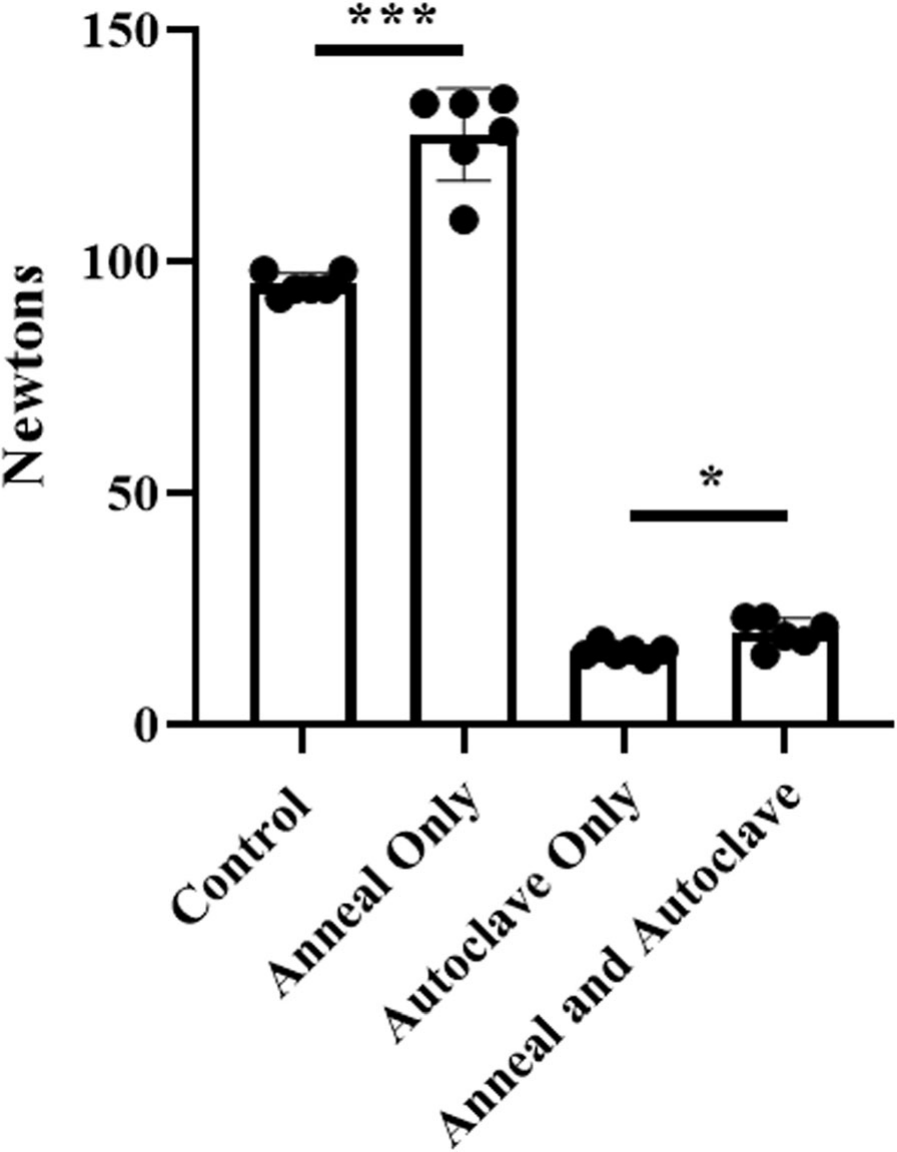
Standard Army-Navy retractor strength across interventions

**Fig. 5:**
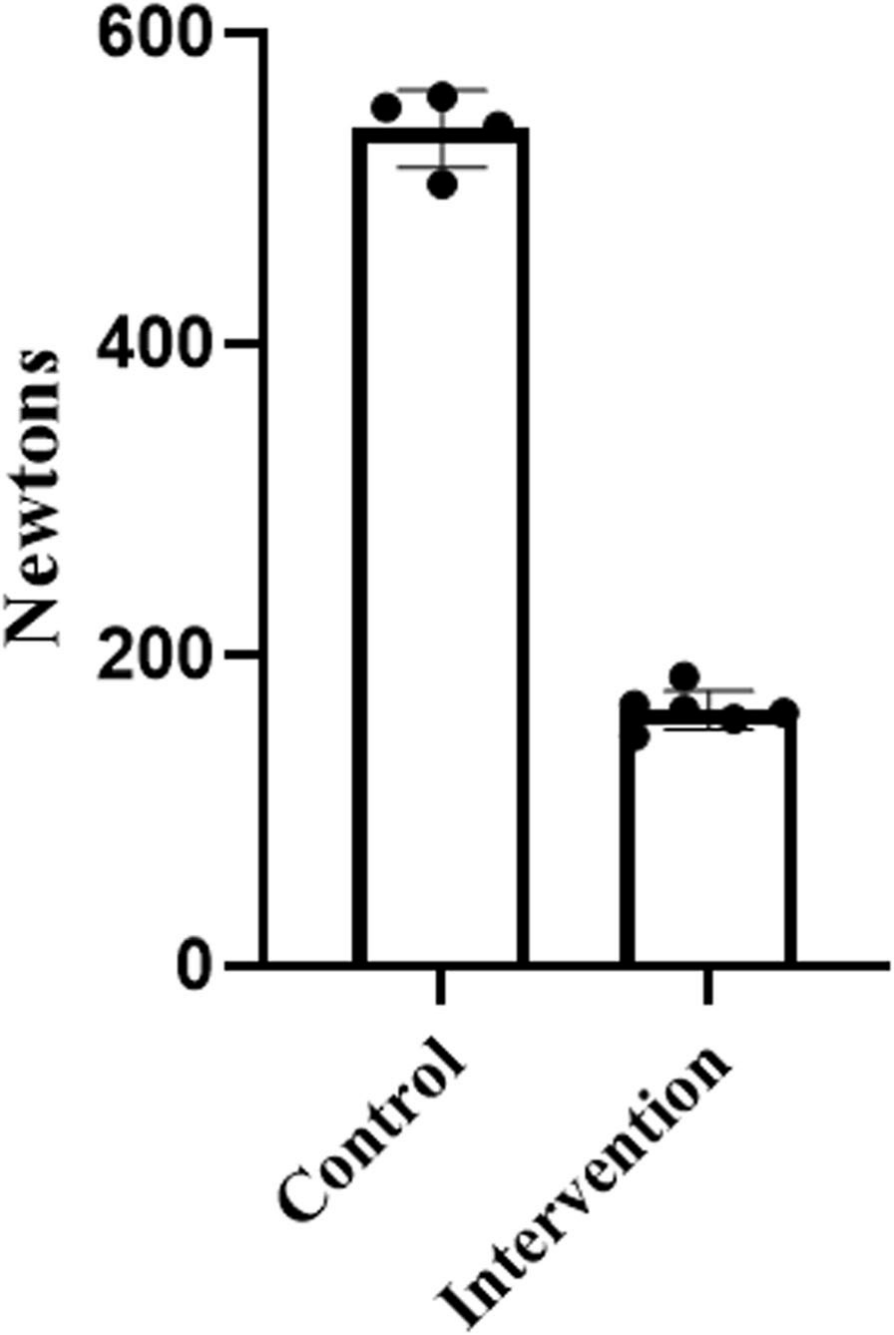
Strength-optimized Army-Navy retractors decrease substantially in strength after annealing followed by autoclaving intervention

Standard retractors without intervention failed at 95 N +/− 2.4 N. Hot water-bath annealed retractors failed at 127.3 N +/− 10 N. Autoclave only retractors failed at 15.7 N +/− 1.4 N. Hot water-bath annealed then autoclaved retractors failed at 19.8 N +/− 3.1 N. Two-tailed T-tests were used to assess for statistically significant differences in strength between these groups. Statistically significant differences in retractor strength were found between the control group and the annealed group (*p* < 0.0001), between the control group and the autoclave only group (*p* < 0.0001), and between the autoclave only group and the annealing then autoclave group (*p* = 0.0135) (Fig. 4). Strength-optimized retractors, after the annealing then autoclaving intervention, failed at 164.8 N +/− 12.5 N (Fig. 5). For comparison, these retractions fail at 538.5 N +/− 24.9 N without intervention. Retractors displayed mild warping after hot water-bath annealing (Fig. 3).

## Discussion

### Optimal commercially available 3D printing material and infill geometry for medical use

From the variations tested, the most optimal 3D printing material for 30 mm cubes was Essentium PLA and the most optimal infill geometry was “grid”. This material and infill geometry consistently deformed the least after hot water-bath annealing and subsequent autoclaving. As such, this combination of material and infill geometry could be a candidate for printing anatomical models.

We identified the Maker Series PLA White HOT White to be the least optimal 3D printing material due to excessive and uneven deformation, making it difficult to preemptively correct for in the STL and print-settings. The least optimal infill geometries are “zig-zag” and “tetrahedral” due to substantial deformation after treatment.

The distortion from heat treatment occurs primarily in the Z direction, the axis perpendicular to the build plate, nearly twice the magnitude of either the X or Y direction. We suspect that disrupted adhesion between deposited layers plays a role in this increased deformation. This information may be used to adjust layer height to account for distortion in anatomical models when preparing the STL.

### Effect of annealing and autoclaving on 3D printed PLA Army-Navy retractor strength

We have found that hot water-bath annealed standard 3D printed PLA Army-Navy retractors demonstrate a statistically significant increase in retractor strength as compared to no intervention. Conversely, autoclaving these retractors substantially decrease their mechanical strength, rendering them extremely weak and brittle. Combining these interventions, annealing then autoclaving, results in retractors slightly stronger than autoclaving alone but still substantially weaker than the control retractors. Therefore, it is demonstrated that annealing 3D prints increase their strength, regardless of an additional autoclaving intervention. However, for the print technology, materials, and autoclave cycle chosen in this study, autoclaving poses a challenge for adopting 3D printed surgical instruments in the operating room by introducing strength limitations. We found that the strength-optimized 3D printed Army-Navy retractor design did overcome this strength limitation.

Using Chen et al.’s definition of clinically excessive retraction to be 35 N, standard 3D printed Army-Navy retractors after annealing then autoclaving do not meet the demands of the operating room, failing on average at 19.8 +/− 3.1 N. However, strength-optimized retractors after annealing then autoclaving fail at 164.8 N +/− 12.5 N, approximately 4.7 times what is needed for excessive retraction. Despite the substantial weakening of retractors due to autoclaving, optimized retractors can survive autoclaving and remain robust enough to use in the operating room.

Strength-optimized retractors without intervention fail at 538.5 N +/− 24.9 N. After annealing then autoclaving, these retractors retained 31% of their original strength. Standard retractors decreased in strength from 95 N to 19.8 N after annealing then autoclaving, resulting in retractors that retained only 21% of their original strength. Therefore, there is not a consistent percentage decrease in retractor strength across different retractor designs.

### Limitations

The results of this study are only valid for FDM 3D printing technology. Our analysis focused only on testing seven commercially available FDM 3D printing materials based on market availability and we tested only four of nine infill patterns available in the Ultimaker Cura LulzBot Edition software. Therefore, there may exist a combination of 3D printing material and infill geometry that is further optimized towards minimizing model distortion after annealing and autoclaving. There may also be alternative methods of sterilization that further minimize model distortion including sterilization using ethylene oxide gas or gamma radiation.

We acknowledge that dimensional changes and strength limitations may not be a challenge at a lower autoclave cycle, which would require further testing. We have also yet to understand the mechanical behavior of the 3D printed models in this study when they are subjected to multiple cycles of autoclaving and whether they will continue to undergo dimensional change. However, regardless of whether 3D printed PLA surgical instruments are determined to be single or multi-use, these instruments may still be valuable in fields such as aerospace medicine where space limitations exist, or in resource-limited situations where additional instruments are needed.

During annealing interventions, 3D prints in this study came in direct contact with hot water during the hot water-bath annealing process. However, if 3D prints were bagged and sealed prior to submersion during the annealing process, dimensional change and strength may have been differently affected. Furthermore, hot water-bath annealed retractors demonstrated warping, which likely introduced random error into our measurements. 3D printed retractors in this study were buoyant in the hot water-bath, and were fully submerged by placing them into a large glass beaker. This positioning of these retractors may have contributed to additional warping, as according to the manufacturer technical data sheet, the heat deflection temperature of Essentium PLA is 70 °C [28].

In this study, retractors were immediately autoclaved after annealed prints were brought to room temperature undisturbed. In the operating room, there may be a difference in time frame between annealing and autoclaving that may also present different dimensional and strength changes.

## Conclusion and future steps

As the field of medicine begins to adopt 3D printing technologies, understanding the mechanical behavior of 3D printing materials becomes critical. Identifying an FDM 3D printing material that deforms the least under heat treatment is important for clinical applications as deformation can change the structural integrity and functionality of 3D printed models and surgical instruments. This study identified an FDM 3D printing material and infill geometry that minimizes 30 mm cube deformation after hot water-bath annealing and autoclaving: Essentium PLA and “grid” infill geometry.

We demonstrated that hot water-bath annealing results in markedly increased 3D printed retractor strength and that sterilizing 3D printed objects using an autoclave drastically diminishes strength. Despite this, the optimized 3D printed PLA Army-Navy retractor design overcomes this strength limitation. 3D printed objects can withstand autoclaving with minimal distortion and maintain model integrity when mechanical strength is not needed, which is helpful for surgical anatomical models used as reference objects in the operating room. Reducing variability among prints is an important step for the continued adoption of 3D printing technology in the medical field and applying it to the creation of low-cost surgical instruments and medical equipment in space and resource-scarce settings, improving healthcare globally. Among future steps in reducing variability, the behavior of alternative low-cost 3D printing technologies including stereolithography 3D printing requires further investigation.

This study is intended as a pre-clinical evaluation of the mechanical behavior of FDM 3D printing materials following hot water-bath annealing treatment and autoclave sterilization. For FDM 3D printed Army-Navy retractors, further sterilization and biocompatibility validation will be necessary for it to be applied clinically.

## Supplementary information

**Additional file 1.** Supplementary data.

## Data Availability

Retractor STLs and data are available through the authors.

## Abbreviations

FDM: Fused deposition modeling
HTPLA: High temperature polylactic acid
PLA: Polylactic acid
STL: Standard tessellation language
